# Defining discovery: Is Google Scholar a discovery platform? An essay on the need for a new approach to scholarly discovery

**DOI:** 10.12688/openreseurope.14318.2

**Published:** 2022-06-07

**Authors:** Kelly Achenbach, Marta Błaszczyńska, Stefano De Paoli, Francesca Di Donato, Suzanne Dumouchel, Paula Forbes, Peter Kraker, Michela Vignoli

**Affiliations:** 1 Max Weber Stiftung – Deutsche Geisteswissenschaftliche Institute im Ausland, Bonn, 53173, Germany; 2 Institute of Literary Research, Polish Academy of Sciences, Warsaw, 00-330, Poland; 3 Abertay University, Dundee, Scotland, DD1 1HG, UK; 4 Istituto di Linguistica Computazionale “A. Zampolli”, Consiglio Nazionale delle ricerche (CNR), Pisa, 56124, Italy; 5 TGIR Huma-Num, CNRS, Aubervilliers, 93300, France; 6 Open Knowledge Maps, Wien, AT-1060, Poland

**Keywords:** Open Science, Content discovery, Information seeking, Social Sciences, Humanities, Infrastructure, Search engines, Multilingualism

## Abstract

This essay discusses the concept of discovery, intended as content discovery, and defines it in the new context of Open Science, with a focus on Social Sciences and Humanities (SSH). Starting from the example of Google Scholar, the authors show that this well established service does not address the current needs, practices, and variety of discovery. Alternatives in terms of technical choices, features, and governance, do however exist, offering richer and more open discovery. The paper presents in particular the implementations and research work of the H2020 project TRIPLE (Transforming Research through Innovative Practices for Linked Interdisciplinary Exploration). Dedicated to the building of a discovery platform for the SSH, the project is meant to address the specificities and evolution of discovery in this field.

Prevailing scholarly resource platforms like Google Scholar limit discovery by focussing only on publications, and favouring through their algorithm well-cited papers, English content, and discipline-specific resources. A limitation in the context of cross-disciplinary and collaborative Open Science, such a service more specifically hinders discovery in the SSH. Characterized by a fragmented landscape, a variety of languages, data types, and outputs, research in the SSH requires services that fully exploit discovery potentialities.

Moreover, a survey conducted within the TRIPLE project showed that most SSH researchers use Google Scholar as their starting point, and that they recognise the lack of control they have with this system. Beyond the extension of features and content, transparency is the other important criterion for the building of an Open Infrastructure actually serving the research community. In light of this, we present in some detail the GoTriple platform, which exploits today’s technological potential and incorporates the best known functionalities in order to unveil more and innovative scholarly outputs and lead to international and interdisciplinary research project collaborations.

## Plain language summary

This article considers how researchers can find articles, books, or data on the web. The number of research materials available online has increased and it is therefore more difficult to find the useful ones. The existence of various platforms makes it even more difficult because they don’t contain all the information and make their own selection of the research material that they present. Some platforms tend to choose materials that are useful only to the majority. This is the case especially with Google Scholar, a platform very similar to Google, but for researchers. Google Scholar only contains articles and books, not data. It also contains mostly publications in English and publications that are the most popular. This is an issue for some scientific disciplines, especially for social sciences and humanities. These disciplines don’t always use English in their work and publications. Their journals and book publishers are not as popular as the others. For this reason, the researchers have difficulties finding useful articles, books, or data on Google Scholar and other platforms.

In our article, we present another way to build a platform that could actually help the researchers of social sciences and humanities. The GoTriple platform makes it possible to find articles, books, or data in these disciplines. It also has content in many languages and allows users to make searches in nine European languages. Furthermore, GoTriple has additional services that help the researchers to easily find appropriate content for their research.

## Introduction

The goal of this paper is to reflect on the concept of discovery and in particular to offer a definition of this concept. It should be noted that we are not talking about scientific discovery itself, i.e., the production of new knowledge. Rather we focus on the way those who are not involved in the production of this knowledge come to know of it, during or after the new knowledge is produced. We do so by reflecting on some of the results of the research project TRIPLE, which aims at building a discovery platform, called GoTriple, for Social Sciences and Humanities (SSH) research. We reflect initially on the limits of the platform, Google Scholar (GS), which has become a key point for academic discovery. Further, we argue why today open infrastructures have become the strongest driver of innovation in discovery.

When GS launched in 2004, it also brought Google's trademark search interface with it, which was considered a major improvement over that of previous search engines. While this interface was certainly innovative at that time, Google has since stopped integrating the new developments from its general web search into GS. In fact, it has been shown that the interface has hardly been updated in the last ten years ( Kraker *et al.*, 2021). This means that Google has not invested enough in GS to keep up with the growing research output. The search results list, which presents only ten items at a time with very limited context, and just a few filter and sorting capabilities, is not well-equipped to provide an overview of the hundreds of millions of research papers in existence.

Additionally, GS only indexes scholarly articles and books in the strictest sense. Even common article types such as editorials and book reviews are out of scope ^1^. This means that while previous studies suggest better coverage for certain disciplines through GS content from disciplines with a high biblio-diversity such as social sciences and the humanities is more difficult to discover and remains sometimes hidden from GS users. It also means that additional output types from today's open science ecosystem, such as images, software, and datasets, cannot be found via GS. As such, GS lacks the ability to produce results that include relevant output and services from higher education institutions. This is a significant limitation because research outcomes beyond scientific publications are playing an increasing role in the context of digitisation of science ( European Commission, 2016). In several user studies on discoverability needs of SSH researchers done in context of the TRIPLE research project, participants confirmed that for them it is not only important to find relevant and current research publications, but also to be able to find data in various formats as well as outputs associated to both past and on-going research projects.

Furthermore, Google does not allow third parties to reuse or republish the GS index, and it has technical means in place to prevent users from crawling their content with automated means. As such, GS’s data cannot be considered to be an open infrastructure, as the data is not reusable as defined in the FAIR principles ( Wilkinson *et al.*, 2016) and the service is not part of the Internet of FAIR Data and Services (IFDS) ( Mons, 2019). The IFDS is the network of FAIR data repositories and other FAIR services connected together through the internet technologies. It requires machine-actionable data and accessible services that GS does not provide.

In comparison, content aggregators from the open discovery infrastructure such as OpenAIRE and BASE provide reusable, machine-actionable and transparent databases over hundreds of millions of scholarly resources of many different output types. These services are also transparent in the sense that they clearly state their sources and offer institutions the ability to add their own content without restricting, for example, the language in which an output was written. This contrast shows how far GS has fallen behind when it comes to equity and transparency.

Overall, while GS does seem to offer some marginally better equality than citation databases, it is clear from previous literature that the service presents several pitfalls. The lack of transparency and openness and the ranking algorithm in particular, appear as major stumbling blocks to consider GS as a complete discovery service ( Beel & Gipp, 2009). It appears therefore necessary to go back to the discovery fundamentals and clearly assess the current needs and potentialities in the context of open science. The design of discovery platforms has to correspond to a renewed and more comprehensive definition of discovery. The paper will hereafter describe the GoTriple platform, which offers one example of such a redefinition, under both the technical and the governance aspects.

## Why Google Scholar is not a complete discovery platform

With the increased availability of materials in digital form, identifying specific resources has become at the same time both easier and more difficult. While clearly scholars have many more opportunities to access material than before the advent of digital technologies, as Cahoy (2018) says ‘Yet as the Web (and information technology in general) has matured, finding and retrieving information has become more fragmented. Users previously had two choices not so long ago—get it from the shelf or find it electronically via a database’. Traditionally, the main location for the identification of scholarly resources was the library, with its collection and catalogues. However, new digital technologies have brought changes to the way these operate ( Favaro & Hoadley, 2014), and now the main sources for identifying resources have become others. We have witnessed originally the emergence of citation databases, largely supported by editors, such as Scopus or the Web of Science (WoS). These operate largely by indexing publications. For some authors, these sources still remain “the main sources for citation data” ( Mongeon & Paul-Hus, 2016).

An important innovation in this area has been, however, the introduction of GS. Unlike citation databases (such as Scopus or WoS), GS is a search engine that operates similarly to Google. According to Lopez-Cozar *et al.* (2019) it ‘represents a break from this paradigm. Unlike traditional bibliographic databases, which are selective by nature, Google Scholar parses the entire academic web, indexing every scholarly document it finds regardless of its quality, and doesn’t differentiate between peer-reviewed and non-peer-reviewed content’. There are indeed several studies which have tried to assess the differences between the ‘traditional citation databases’ and GS for the identification and retrieval of resources (e.g. Harzing & van der Wal, 2008; Ştirbu *et al.*, 2015). Research has been conducted also to compare GS with open access repositories such as OpenAire and others ( Abad-Garcia *et al.*, 2018). Generally, there is no consensus on whether GS is a valid alternative to scholarly citation databases. For some authors (e.g. Haddaway *et al.*, 2015) GS is not a valid replacement of services like WoS, where the former has been seen as missing relevant literature in the conduction of systematic analysis. Other authors, ( Harzing & Alakangas, 2016) instead support the notion that GS offers a broader coverage of material than databases.

Notwithstanding the debate on the differences, advantages and disadvantages of GS over other services for conducting systematic literature analysis, it remains the fact that GS has become a main port of call for many literature searches ( Kramer & Bosman, 2016). It is a simple and free to use service and many start their literature search directly from GS. For instance, for Jensenius *et al.* (2018) says ‘the advantages of Goggle Scholar stem primarily from its ease of use.’. The question is whether GS can be seen as the main resource for facilitating discovery. While the discussion on the differences in coverage with databases (Scopus, WoS) concentrates largely on the scholarly efficiency, other aspects need to be considered in the discussion.

Several authors have compared GS with citation databases in other areas, for example on languages coverage, identification of grey literature, open access, disciplines, and diversity more generally. Whilst it may be argued that academic databases could provide some edge for systematic literature retrieval, much literature converges on the idea that GS also fares better in this area than the academic databases. For example, GS also offers profiles for users, and the metrics contained therein have been argued to ‘offer grounds to challenge unfounded perceptions and prejudices’ ( Jensenius *et al.*, 2018) that are widespread in research, for example, affecting women or minorities. In terms of language coverage, Lopez-Cozar *et al.* (2019) have shown empirically that ‘while the percentage of documents published in English in WoS and Scopus is of 90% and 80% respectively, in Google Scholar the percentage is closer to 50%’, concluding that GS offers better representation of languages other than English, when compared with academic databases. This was also noted previously by Jacsó (2008). An empirical investigation on the coverage of Chinese publications in GS and other citation databases shows that GS coverage is better than other services ( Zhang & Lun, 2019). This edge on the language coverage is of course due to the nature of the service offered by databases, which are products of academic private publishers that largely have publications in English, while GS, as a search engine, harvests the entire web. This also leads to better open access coverage than citation databases, as indeed, GS can also harvest open access versions (e.g. pre-prints) of papers that otherwise are not accessible because they are behind paywalls ( Jensenius *et al.*, 2018). In addition, GS has agreements with publishers to even gain access to closed access items for which no open access version exists ( López-Cózar *et al.*, 2017). Finally, authors have argued that GS seems to offer better coverage of certain disciplines if compared to citation databases, in particular but not only, for social sciences ( Harzing & van der Wal, 2008).

Overall, while the literature paints a picture of GS as a service more “equitable” than citation databases, it is difficult to assume that it offers a full and transparent solution. Although offered for free, GS still remains a closed service to a larger extent ( Coiffait, 2019; Kraker *et al.*, 2021). Indeed, several authors have pointed to the lack of transparency offered about how the service algorithms work, which to a large extent mirrors what happens with the main search engine Google (e.g. Rovira *et al.*, 2021). Other authors have pointed out the secrecy of the sources, from which GS retrieves the publications; ‘There is no information about the publishers whose archive Google is allowed to search, let alone about the specific journals and the host sites covered by Google Scholar’ ( Jacsó, 2005). This notion was supported also by Harzing & van der Wal (2008). Thus, the lack of openness about the algorithms and the sources used by GS makes this service unlikely to be the exemplar of equality and transparency.

Despite the earlier noted better language coverage of GS if compared to citation databases, studies have also shown that GS still remains skewed towards English. This is especially due to the ranking algorithms, which perhaps is one of the components of the service that creates more inequality. The ranking algorithm puts the most cited papers at the top of the search results, and these normally tend to be publications in English: ‘the ranking algorithm favors documents in English and handicaps documents written in other languages in searches that produce multilingual results’ ( Rovira *et al.*, 2021). The ranking algorithm of GS has been seen as a source of problems and inequality in other areas as well. Since citations have a huge influence on the ranking, some authors have argued that this facilitates incremental research over very innovative publications: ‘Highly original work that does not fit neatly into an existing literature might establish a new research agenda and expand interest in the topic, but its impact will not be visible in citation counts for many years’ ( Jensenius *et al.*, 2018). Likewise, the ranking algorithm tends to favour the visibility of well-established scholars over others, which may have a significant impact, especially for early careers ( Jensenius *et al.*, 2018). When we specifically consider the relationship between GS and SSH, there are few research publications which have focused on understanding how well this search engine serves SSH. These publications tend again to offer a comparison between GS and established citation databases (e.g. Scopus) and pretty much highlight similar issues as those discussed earlier. Gardner and Eng (2005) (in a perhaps not very recent paper) had shown that differences exist between GS and repositories/databases such as PsycINFO or the Social Sciences Citation Index, for retrieving social sciences literature on a specific topic (“homeschooling”). While GS appeared to offer a large variety of sources in relation to a specific research topic, it also appeared to retrieve publications which possess less academic “currency” than those that can be retrieved with established databases. Of course, the algorithm has changed a lot since this article. And it heavily relies on citations for ranking now. However, Prins *et al.* (2016) have conducted a similar comparison, however focusing on the use of GS and other databases (such as Scopus) for the conduction of formal assessment/evaluation of research programs, with a focus on humanities (e.g. Anthropology). The authors concluded that GS could be used for such evaluations, but differently from repositories, the material retrieved by GS requires assessing the quality and whether it is reliable. From these two sources, it appears that reflection on the use of GS in SSH does concentrate much on the differences with other established databases in establishing what counts as relevant publications in the SSH broad area. Similarly to what seen earlier, GS does appear to offer a broader variety, thus supporting potentially the retrieval of less main-stream material, which however for purposes of e.g. evaluation, systematic analysis may be less relevant or anyway requires much work for filtering out what is established content and what is not. This point of difference was raised, with a different spin, also by Martin-Martin *et al.* (2019) which, looking at the use of GS, Scopus and WoS in SSH, pointed that GS offers an approach which is more “inclusive and unsupervised” if compared to the “selective” approach of the databases Scopus and WoS. Indeed, whilst the latter pretty much index mostly journal papers, GS also includes a variety of other sources such as dissertations, book chapters etc. This last contribution thus seems to argue for the approach of GS as being more inclusive, however warning that there is a trade-off, since we would then not be working with well-curated databases.

Should we conclude then that GS better serves SSH in terms of inclusivity of sources? (even if the price to pay is less curation?). Whilst this appears potentially true from the few analyses we have, as cited above, it is arguably also coming with its own limitations. We have already pointed at the issues around language, the opacity of the algorithm, the fact that GS comes as a top-down solution based on the google approach and so on. A discovery service oriented specifically at SSH should be even more “inclusive and unsupervised” than GS, potentially offering a level playing field for languages (i.e. treat all languages with the same importance). Moreover discovery should not just be limited to publications and should encompass the possibility to discover other relevant information such as projects, people or funding sources. Thus an SSH discovery solution should broaden the scope of discovery, to foster aspects such as community building and wider collaboration. Additionally an SSH discovery service should be directly designed together with the SSH community, via codesign and direct analysis of needs and pain points. That is, it should be built from the ground-up by working with the SSH community in order to have a discovery system that adapts well to different needs and contexts of work. This is of course not the case for GS that, despite its excellent usability, comes as a top-down solution. All these novel aspects are what the TRIPLE research project aims to achieve, with the design, development and release of the GoTriple platform.

In light of GS's limitations the TRIPLE project built a discovery platform, GoTriple, which aims to provide a service to discover SSH resources (data & publications, profiles of researchers and scientific projects) with a multilingual perspective. By linking these resources together in one platform, the user's experience and the ability for researchers and society at large to discover resources for their specific purposes should be much improved. The TRIPLE project has worked with stakeholders, from across SSH disciplines and wider, from the very start to ensure that the platform will take into account their needs for a discovery platform. In doing so, all aspects discussed throughout this paper that constitute effective discovery platforms have been taken into consideration. Moreover the GoTriple platform enables users to discover and reuse open scholarly SSH resources in nine European languages which are currently scattered across local and discipline-specific repositories, it also provides opportunities for researchers and projects to connect across disciplines and languages. A set of innovative tools and services are integrated in the platform to support research and enable, among others, the visualisation of search results, web annotation, personalised recommendations and social networking practices. In line with the aim to connect researchers and projects in the SSH, the platform allows users to explore new ways of funding research such as crowdfunding. The presentation of the GoTriple discovery platform and its features will be deepened further in the article (see section 4). 

## What is discovery?

In this section, we take a step back to reflect on the meaning and process of discovery—and in light of the research on GS cited in the previous section, it becomes clearer why researchers need more than what GS can offer to facilitate their discovery process. The discussion begins with some background information describing the TRIPLE project, its aims and its methodology for facilitating the discovery process in SSH.

The TRIPLE (Transforming Research through Innovative Practices for Linked Interdisciplinary Exploration) project was funded by the European Commission under the framework of the Horizon 2020 program, as an innovative service for the European Open Science Cloud. SSH research is divided across a wide array of disciplines, sub-disciplines, and languages. While this specialisation makes it possible to investigate the extensive variety of SSH topics, it also leads to a fragmentation that prevents SSH research from reaching its full potential. Use and reuse of SSH research is suboptimal; interdisciplinary collaboration possibilities are often missed, and as a result the societal, economic and academic impacts of SSH remain limited. The TRIPLE project seeks to address these issues, and the resulting platform, GoTriple, is designed and developed to address the discovery issue in the SSH.

The TRIPLE project methodology is based on the combination of two complementary approaches: The first one is the complete adoption of Open Science practices for the design and development of the platform; and the second one refers to the adoption of a user centred approach focusing on understanding the researcher needs and co-designing some core aspects of the platform with them. The decision of the TRIPLE project to adopt the term “discovery” to define a search platform for SSH resources, reflects the complexity of this endeavour, especially considering SSH specificities, in terms of multilingualism, fragmentation, bibliodiversity—all issues which couldn’t be properly addressed by a traditional search platform.

‘With the term discovery we mean the capacity to explore, find, access and reuse material such as literature, data, projects, researchers' profiles etc. that you would need for your own research work (such as finding a relevant paper which will help with your research, or finding a person you are interested in collaborating with)’ ( Pohle *et al.*, 2020).

A common discussion on the meaning of “discovery” has been part of dedicated activities throughout the duration of the project. For example, 37 qualitative interviews with end-users (SSH European researchers) and other stakeholders across Europe, were run during the period December 2019 - March 2020 ( Forbes *et al.*, 2020).

The core themes identified give some general views about SSH’s quite heterogeneous working practices. In the initial discovery phase, the majority of SSH researchers use Google Scholar as a search method. A considerable number then consolidate a Google Scholar search by looking at other academic databases. Some academics also use Twitter as an information source. Most issues affect the effective findability (not finding everything that you need when searching, especially difficult in interdisciplinary research, with a silo effect of research being published in specific disciplines which makes it more difficult to discover if the research is not so rigidly defined; differing keyword terms for similar topics used by different disciplines makes it harder to search; language differences when searching - different terms being used in different languages) and reusability (including the impossibility to access full texts, and to export resources in different formats).

As part of the interview, interviewees were asked to express their desired features for a new platform to ease their discovery work practices and ultimately facilitate their research work. When asked what functionality could perhaps make their life easier, academics replies focused on two main topics:

- Enable establishing connections and collaborations among researchers, finding proper networks and creating community practices for specific topics.
- Explore new functionalities, both widening the possibility to retrieve results other than publications (personal profiles and projects) and through visualisation and recommendation systems.

More recently, in May 2021, TRIPLE project organised an online ThatCamp ^2^ with a focus on “ Discovering discovery”. The ThatCamp was organised as a forum to share ideas, strategies and resources for discovering research and getting research discovered. Eight sessions of 90 minutes each were open to discussion and brainstorming around specific questions/topics; which were nominated and voted on by the participants themselves.

Starting from some general questions, such as:

- How would you define “discovery” in the research cycle?
- What is your epistemological stance on “discovery” in a research context?
- What are your current strategies for discovering research resources?
- How do you make sure your own research gets discovered?
- What are you struggling with technologically?

Each session ^3^ was then dedicated to discussing specific aspects related to the discovery and reuse of open scholarly SSH resources, i.e., research data and publications, across disciplinary and language boundaries; to find and connect resources with other researchers and projects; to make use of innovative tools to support research; and to discover new ways of funding research ( Pohle *et al.*, 2021).

With each session covering slightly different issues and perspectives in relation to discovery, the focus of the discussion differed from group to group. However, there were certain common themes identified, which reflect and confirm the views that emerged from the qualitative interviews. These can be grouped into two types: first, theoretical reflections on what discovery means and what the process of discovery entails in the scholarly context; second, practical implications on what characteristics and functionalities the discovery platform built within the TRIPLE project should have. We discuss these aspects in the section below.

*What does discovery entail? Theoretical reflections.*

During the ThatCamp session devoted to understanding the meaning of ‘discovery’ in different languages, it emerged that the concept is not easily translated. The participants considered the meaning in seven different languages: Croatian, French, German, Italian, Portuguese, Serbian, and Slovenian. Discovery has many connotations and there seems to be a pressing need for discussing its different facets. Three specific aspects of discovery that invited further exploration were *finding what was not expected*; *finding by linking together*; and *finding meaning*.

While ‘finding’ might suggest that we have a set goal and know what we are looking for, ‘discovery’ implies a richer journey of resource exploration—one that can be ensured by the richness (and correct use of) metadata, especially topically relevant keywords. A paper, a dataset or a researcher profile may lead us to several other materials. Thus, the keywords need to be broad yet specific - discovery should not imply browsing through an endless list of materials that are only marginally linked to our topic of interest. Moreover, *finding what was not expected* can mean the access to work in different languages (especially the ones we do not speak or do not normally use in the academic context), broadening the range of perspectives. 

Through *finding by linking together* we understand the interconnection between different kinds of resources that would often exist separately on more traditional search platforms. In discovering, more so than in conservative searching, one type of record can easily lead us to another. A dataset might be linked to its author, whose researcher profile will in turn bring the user to a relevant publication. Here discovery implies the relatively easy possibility to uncover various materials that are somehow related.

*Finding meaning* implies not simply uncovering new resources but also interpreting them, applying a deeper understanding to their value. When discovering, one does not simply see a new object but also comprehends it and can decide whether or not it will be of use in the context of her research.

There was also a need to draw a distinction between the ‘discovery’ as the final end such as a paper or a dataset that has been discovered - *the ‘object’ of the discovery* - and the definition of ‘discovery’ as the set of practices (e.g. existing explicit and implicit skills) and tools (e.g. search engines, recommender systems, multilingual tools), that used together allow a researcher to discover something—that is *the process of discovery*. In this context, tools are not just means to the end, they also play an important role in the process and have an agency on their own that may very well also include biases. Thus, discovery as a process is a *network*.

*What do researchers need from a discovery service? Practical considerations.*

Many of the reflections and comments shared by the TRIPLE ThatCamp participants referred to their everyday practices of discovering research and the challenges related to this process. When discussing the difficulties in discovering, several issues were raised during the workshop, including the following:

- Differences in terminology across SSH disciplines and also languages
- Conflict between focussed searching and serendipitous discovery
- Unknown ‘ranking’ of articles by the algorithms operating in the ‘black box’ of the recommender system
- Difficulty in finding projects (especially after they are completed)
- Difficulty in finding articles based on keywords - due to a lack of linkages
- Time delay between articles being published and them being listed in discovery systems
- Lack of access due to paywalls
- Not being able to distinguish between Open Access and articles unavailable due to paywalls (lack of clear labelling)

Participants also mentioned that they would prefer to have a link to the original landing page where the article was submitted, not just a link to the PDF file (this is especially useful for viewing supplementary material and is also important for tracking the number of downloads of articles which the author needs to know for impact metrics). Participants were keen to have more control over what’s important to them (for recommender systems and search algorithms). There was debate over the extent to which users should be guided or left free to make their own choices on discovery.

The discoverability of relevant tools to analyse data and other software was mentioned as being problematic, GitHub is often used as a community resource, but this does not mean that the software is particularly discoverable.

In another session, it was also noted that in some platforms or repositories, the researcher also has the opportunity to influence the discoverability of her work. This role is often overlooked but in many instances, it is the researcher who delivers the metadata, such as keywords, to the repository. While the average researcher will be the one with the academic expertise and best appointed to explain the value of a paper they have written, their knowledge of cataloguing systems might be limited. Therefore, there is not just a need to enhance the discovery experience of individuals but also to build a community of researchers, librarians, and technologically skilled individuals, allowing them to collaborate and learn from each other, through the work of liaison individuals or by participating in common projects, training and activities. Thus, a successful platform should not just be an online space for discovering resources but also needs to encourage community-building.

Last but not least, one of the sessions directly addressed the obstacles and challenges in offering a discovery service and how they apply to Open Access resources. Challenges from the library and OA perspectives were explored. Some of the topics were recognised as of crucial importance, for example: the value of preprints (whether they should be included in the discovery platform), the issues surrounding ensuring high quality of the metadata of resources, stability of document handles (such as DOI), indexing, ranking and classification of resources, searching and saving options.

The input from the TRIPLE project interviews and ThatCamp discussed above, suggest that in order to distinguish itself from the big commercial players (e.g. GS), a complete discovery service would need to respond to the needs of researchers and face the challenges listed above. This includes addressing the issue of preprints. There are different opinions on how valuable preprints are, and whether they should be included in a discovery service; often the importance given to preprints varies by discipline. The quality of metadata is also an important issue. High quality of metadata allows one to discover, in the sense of finding meaning (see the section above). A good description of a resource means that one can better understand the context in which it was produced and its relevance to the search. It also allows for interoperability with different databases and data providers. However, since many providers apply different standards, ensuring a common workflow in relation to metadata may be a major difficulty for a discovery service aggregating resources from a number of places. Related to this, is the issue of stability of handles that might break or change. Finally, indexing, ranking and classification of resources should be at the centre of the process. With the current rate of knowledge production, it is difficult to keep track of existing research, even within one’s niche; exploring important literature in a new field proves even more challenging. Filtering the results without narrowing down the discovery process too much is one of the main principles. While they should not be overwhelmed with the resources, users ought to be able to search in an open manner that would ensure that the process of finding what was not expected can also occur.

## Discoverability crisis issues: Why we need an open and inclusive discovery platform

Shifting from the more abstract notions of discovery covered above, we now turn to a concrete and critical example - research on the COVID-19 pandemic - to illustrate the urgency of this matter, followed by a discussion of the overall state of research discovery and an overview of the trajectory of recent developments in research discovery systems. The coronavirus pandemic has triggered an explosion of research, with more than 400,000 publications related to COVID-19 published to date ( OpenAIRE, n.d.). At one-point in 2020, scientific output on the topic was doubling every twenty days ( Brainard, 2020; Schmidt *et al.*, 2020). This huge growth poses big challenges for researchers, many of whom have pivoted to coronavirus research without experience or preparation ( Johnson, 2020).

Mainstream academic search engines are not built for such a situation. Tools such as GS, Scopus and Web of Science provide long, unstructured lists of results with little context. These work well if you know what you are looking for. But for anyone diving into an unknown field, it can take weeks, even months, to identify the most important topics, publication venues and authors ( Kraker *et al.*, 2021). This is far too long in a public health emergency. The result has been delays, duplicated work, and problems with identifying reliable findings ( Ioannidis, 2020; OECD, 2020). This lack of tools to provide a quick overview of research results and evaluate them correctly, has created a crisis in discoverability itself ( Kraker *et al.*, 2021). The pandemic has highlighted this, but with three million research papers published each year ( Johnson *et al.*, 2020), and a growing diversity of other outputs such as datasets; discoverability has become a challenge in all disciplines ( Kraker *et al.*, 2021).

The challenges with respect to this information overload are reflected in a lack of reuse of scientific knowledge: depending on the discipline, between 7% and 38% of research papers are never cited, rising to 63% of those without a disciplinary classification ( Nicolaisen & Frandsen 2019). In the case of data sets, the uncitedness even increases up to 85% ( Peters *et al.*, 2016). We can also see effects for reuse in practice; even in application-oriented disciplines like medicine, only a minority of research results are ever applied in clinical practice, and if so, then with a long delay ( Brownson *et al.*, 2006). This suggests that we are in a veritable discoverability crisis, where a large amount of public knowledge remains hidden. This crisis goes far beyond the coronavirus pandemic. In 2019, a group of researchers coined the term *dark knowledge*, which relates to scientific knowledge that cannot be found and reused ( Jeschke *et al.*, 2019). The researchers assume that there is more dark knowledge than there is discoverable knowledge, and that the share of dark knowledge is rising.

These observations are supported by the extensive user studies carried out in the TRIPLE project (see Table 1 for relevant quotes from participants). During the initial interviews with SSH researchers and other stakeholders, difficulty in getting an overview of the research field was a common theme, especially when the topic of research was new to the person trying to make sense of the information, or the topic was interdisciplinary. Although GS is often used as a starting point, it is usually backed up by searching via another method, such as a University library catalogue, Web of Science, Mendeley database, or a specific discipline database. This becomes even more difficult when the topic is interdisciplinary due to the siloed nature of publications and the way that traditional journal publications have become increasingly narrow in focus. Researchers report the need to use many different information sources to try and gain this overview and to ensure that they are able to find all relevant publications or data.

**Table 1.** Paraphrased quotes from the TRIPLE user research ^1^.

*"I work on a narrow topic at the crossroads of art history and history of sciences* *and I have to go through a very wide range of publications over two centuries. It is* *difficult to find what I am looking for because of the broad range of subjects but a* *very narrow focus at the same time."*	***P3 in the workshop*** ***on visual discovery***
*"We have a lot of data available but sometimes this notion is missing that we had* *when we went to the library when a title gave us an idea for our research. Right now* *we are missing the possibility of just starting blind and through the research we get* *insights and ideas of what we can do."*	***P7 in the workshop*** ***on visual discovery***

^1^ TRIPLE consortium, D3.2 Report on co-design of the innovative and new services.pdf, under review, submitted in September 2021.

**Figure 1.**
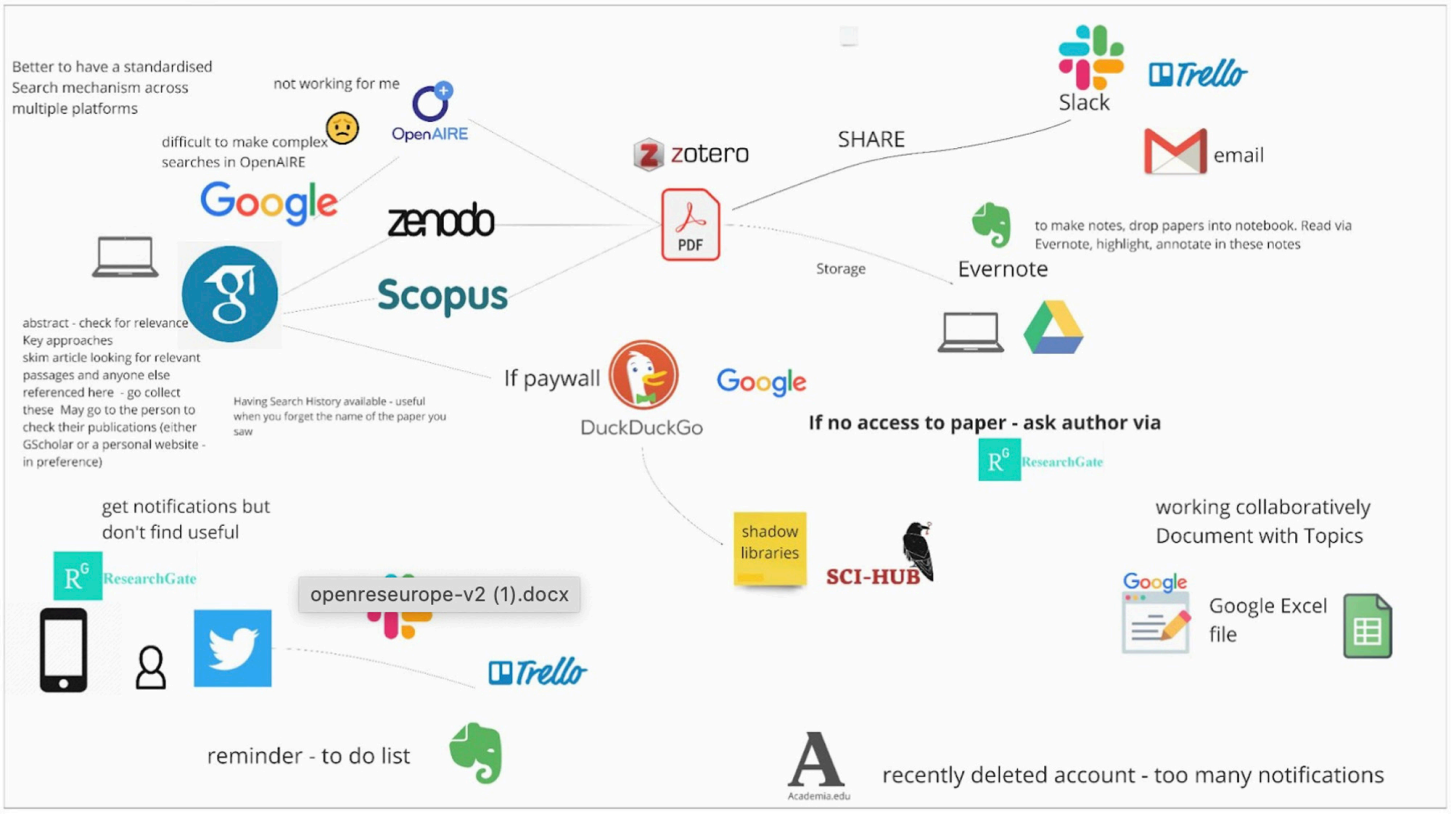
An example of a researcher’s ‘Discovery Journey’ mapped in Miro.

The complexity of a discovery process has been confirmed further in the TRIPLE research by having researchers ‘map’ their discovery journeys and the artefacts that they use for this. An example of a researcher's Discovery Journey is shown below in Figure 1.

One of the reasons for the discoverability crisis is a lack of innovation in discovery systems. For years a few large companies have dominated the market for academic search engines: GS; Microsoft’s recently retired Academic ( Microsoft, 2021); Clarivate’s Web of Science, formerly owned by Thomson Reuters; and Elsevier’s Scopus. But investment hasn’t kept pace with the growth of scientific knowledge. What were once ground-breaking search engines have only been modestly updated, so that mainstream discovery systems are now of limited use ( Kraker *et al.*, 2021). This would not be a problem if others could build on companies’ search indices and databases, but this is usually prohibited (see for example Clarivate, n.d.; Elsevier, n.d.; Google, 2022; ResearchGate, 2020).

This would not be a problem if others could build on companies’ search indices and databases, but this is usually prohibited. This is true for most commercial offerings, even though the business models differ. Elsevier (Scopus) and Clarivate (Web of Science) monetize their services primarily through license fees. Google monetizes Google Scholar in two ways: (1) directly, by including GS results in their web search alongside advertisements and (2) indirectly, by keeping academic users on its platform for their specialized discovery needs. It should be noted that these business models are currently in flux and there is an unfortunate trend in commercial research infrastructure towards surveillance-based capitalism, which builds on data analytics and intrusive tracking of researchers ( Brembs *et al.,* 2020). 

In the shadows of these giants, however, an alternative discovery infrastructure has been created, built on thousands of public and private archives, repositories and aggregators, and championed by libraries, non-profit organisations and open-source software developers. Unlike the commercial players, these systems make their publication data and metadata openly available. Building on these, meta-aggregators such as BASE, CORE and OpenAIRE have begun to rival, and in some cases outperform, the proprietary search engines. Their openness supports a rich ecosystem of value-added services, such as the visual discovery system Open Knowledge Maps, or the open-access service Unpaywall ( Kraker *et al.*, 2019; Kraker *et al.*, 2021).

This open infrastructure has become the strongest driver of innovation in discovery, enabling the quick development of a variety of discovery tools during the pandemic. Technologies such as semantic search, recommendation systems and text and data mining are increasingly available ( Kraker *et al.*, 2021).

Many open systems, though, are not sustainably funded. Some of the most heavily used make ends meet with a tiny core team. Half, including Open Knowledge Maps, rely on volunteers to provide basic services. The funding options for non-profit organisations and open-source projects are very limited. Most rely on research grants, which are meant as a jumping off point, not a long-term solution ( Ficarra *et al.*, 2020; Thaney, 2020). The academic community needs to step up and secure the future of this crucial infrastructure. The shape of research infrastructure depends on institutions’ buying decisions. If most of the money goes to closed systems, these will prevail ( Kraker, 2021).

A first step would be to create dedicated budget lines for open infrastructures. The initial investment would be relatively small, as their membership fees are usually orders of magnitude cheaper than the license fees of their proprietary counterparts. Over time, strengthening open infrastructure will enable research institutions to cancel their proprietary products.

It’s not just about money. Open infrastructures do not lock institutions into closed systems and save them from selling off their researchers’ user data; an issue gaining prominence as large commercial publishers become data analytics businesses ( Brembs *et al.*, 2020). GoTriple goes further than adhering to the latest open data standards. In fact, the Gotriple platform embraces the core principles of a community-owned and community-led infrastructure. An attractive feature of GoTriple is that its users own it, fund it, and direct it (ie. scholars and the public).

The coronavirus pandemic has shown that the challenges of our globalised world demand international collaboration. That requires building on each other’s knowledge. However, this is not possible with closed and proprietary discovery infrastructures that have fallen behind the growth of scientific knowledge. Instead, we need to guarantee the sustainability of the open discovery infrastructure, so that we can rely on it for today’s and tomorrow’s challenges.

## GoTriple: capturing the meaning of discovery platforms

Acknowledging that there is not a one-size-fits-all solution for “discovering” in research, in this final section we introduce the TRIPLE project’s effort to combine the technological and infrastructural developments of the past decade, to find a solution for meeting today’s researchers' needs and desires—the GoTriple discovery service. 

### Inter-language and inter-discipline coverage

GoTriple provides two levels of semantic enrichments in the following nine languages: Croatian, English, French, German, Greek, Italian, Polish, Portuguese and Spanish. The first level relies on the twenty-seven SSH disciplines listed by the European project MORESS (Mapping Research Expertise in the European Social Sciences and Humanities). The twenty-seven discipline titles have been translated in eight languages and a thorough machine learning process made it possible to classify any GoTriple document according to these scientific fields. The MORESS disciplines are also linked with major national controlled vocabularies (e.g., English Library of Congress Subject Headings (LCSH), French RAMEAU or Spanish BNE). The second level relies on the GoTriple thesaurus. The thesaurus was elaborated from the SSH subset of the Frascati taxonomy for research. This subset allowed us to identify broad terms and their children in the LCSH. At the end of this process, the thesaurus gathered 2,565 concepts in English. After using both automated translation and human curation, the thesaurus now contains entries in nine languages, structured in SKOS ( Simple Knowledge Organization System) and linked with Wikidata. The thesaurus is used to provide accurate and interlinked translations of the keywords collected by GoTriple in its harvesting process. Thanks to these two levels of semantic enrichment, the GoTriple platform makes it possible to filter the search and to navigate its results, using either the SSH disciplines (which are broader and come from the MORESS categories), or the SSH topics (which are narrower and can be seen as the subject in a specific discipline) in nine languages. The nine languages were determined by the data and the linguistic skills available within the TRIPLE project, however, the work done has given the necessary expertise to add new languages to the platform in the future. Further monitoring and human expertise will create the possibility to enrich the thesaurus through time with new concepts. This could indeed become necessary to adapt to current events and societal trends (e.g., global pandemic, COVID-19 or teleworking).

### Systematic metadata retrieval

GoTriple aims at referencing a wide range of scholarly resources coming from a high variety of sources in order to ensure biblio-diversity. The platform covers a diversity of disciplines and data types, such as scholarly articles and books, editorials, book reviews, grey literature, images, videos and datasets — all mostly in open access format. The harvesting process relies on a two-step strategy: collecting metadata through aggregators and supporting the data providers, so that they can make their metadata available for the aggregators. This strategy offers an inclusive set of scholarly outputs to the platform’s users, and fosters the alignment of data management activities in the SSH area. GoTriple started collaborations with aggregators that are domain-specific (e.g. cultural heritage) or object-specific (e.g. publications). In addition, we also collaborated with aggregators with a broader scope, working with them to identify in their collections, a subset useful for GoTriple (e.g., using the OpenAIRE gateway’s algorithm to filter SSH contents in OpenAIRE collections). GoTriple is able to harvest metadata from this variety of sources by mapping the various metadata schemas and data models to a single data model. The specific data model is based on Schema.org, a powerful and flexible ontology able to describe the variety of data handled by GoTriple. It is used indeed for the three main types of data that can be discovered on the platform: scholarly outputs, research projects, and researcher profiles. Schema.org allows us to define a rich data model for each of these data types. Moreover, in the case of scholarly outputs, the use of an ontology enables interoperability amongst the multiple data models and metadata standards used by the various aggregators. A rather innovative choice to describe the SSH contents, the schema.org ontology proved to be perfectly appropriate for the purpose of GoTriple.

### Integrated tools that aid in the discovery process

Revisiting the above theoretical discussion, “Discovery” is described as an open and engaging process that includes *finding what is not expected*, *finding by linking together*, and *finding meaning*. For such a multifaceted process to take place, it makes sense that a similarly complex set of tools might facilitate it to open the possibilities of reaching/finding/stumbling upon/creating/co-creating/developing/imagining new and innovative research (not just the incremental research that results from “ranking” algorithms mentioned above). Integrated within GoTriple, therefore, are a number of unique features.

Most prominently, the Visual Discovery System presents search results in multiple new ways (including linking similar articles together in a clustered view), to present topics, ideas and overviews that may not be on a researcher’s one-discipline-focused radar. Based on the open-source framework Head Start by Open Knowledge Maps ( Kraker *et al.*, 2019), the Visual Discovery System can be accessed from the search results page and provide a topical or temporal overview of the user’s query. In addition to the Visual Discovery Systems, GoTriple makes use of diagrams throughout the platform, to provide further insight and context for the data presented.

Additionally, the inclusion of an intelligent and adaptive recommender system provides targeted suggestions to researchers. Recommendations appear in the presentation page of a scholarly resource to point users to similar content, possibly targeted to their specific areas of interest. Moreover, registered users receive recommendations in the Discovery platform homepage for the latest content, relevant to the user, acquired by GoTriple. The recommender system implements several state-of-the-art recommendation algorithms, either based on the textual content of scholarly resources or on the actions performed by users in the GoTriple interface (e.g. documents viewed, preferences set, profile data declared, etc). Additionally, the recommender system aims to implement fairness constraints in its resource ranking procedure, e.g., to boost the recommendation of unpopular, but matching, content (i.e., so-called long-tail resources that are typically underrepresented in recommendation lists).

As mentioned above, GoTriple will contain other innovative services that will be integrated in the final version. The first one is an Open Annotation Tool, to allow users to “take notes” on web resources in the form of highlights, comments and also semantic annotations — that is, formal statements declared as “semantic triples”. A second one is a crowdfunding service for SSH researchers to find financial sources for “small scale” research activities, which can be accomplished with limited investments but need quick timing to be performed successfully. Other third-party applications will be also integrated in the platform, including Bookmarking tools, a Metrics service and a Translation service, for ensuring the presence of an English description for all resources acquired in GoTriple.

Finally, the discovery process is enriched through making connections and having interactions with other researchers and stakeholders. The Trust Building System (TBS) integrated within GoTriple is a social networking service, co-designed with users, that supports users — in a human-to-human, personal way (not only through automated algorithms) — to develop important professional connections within and beyond the research realm for co-creating, collaborating on, finding funding for, and communicating about research projects.

### Quality and transparency of harvested data

The issues of transparency and quality of data are inextricable. Researchers need to know the source of the data they are using to be confident that it is high in quality, which is why the GoTriple platform clearly labels its data sources. Although GoTriple aims to harvest only the highest quality metadata, the challenge remains that data providers do not always offer the necessary level of detail in their metadata for services like GoTriple to achieve this goal. This is why a major component of the TRIPLE project is dedicated to supporting data providers in adapting their data harvesting models, to adhere to the FAIR principles: findable, accessible, interoperable, and reusable. These principles form the foundation of data discoverability.

### Accessibility

A discovery service is only useful if the links and the results it provides are accessible to the user. Many types of scientific data sources are included in the GoTriple results (including links to traditional fee-based journals - which is helpful for those users who have access to them), but a special focus is placed on Open Access content (including publications, data sets and blogs). In this way, GoTriple simultaneously provides its users with useful results and contributes to data reuse in the social sciences and humanities.

### Integration into the larger landscape

Data discoverability must be a whole-system effort. This is why GoTriple is not, and could not, be an independent enterprise, but rather a service embedded within and supported by much larger European research structures. TRIPLE is an OPERAS project; a research infrastructure committed to upholding the FAIR principles as it ‘coordinates and federates resources in Europe to efficiently address the scholarly communication needs of European researchers in the field of SSH’ ^4^. In turn, services developed through OPERAS are included in the European Open Science Cloud Association (EOSC) portal, which was created through the European Commission to provide European researchers and professionals with access to relevant research data. This is the same end goal as GoTriple: to provide resources that effectively support discovering solutions to today’s pressing societal challenges.

## Conclusion

By design, the GoTriple platform is made with open source tools and can be adapted to different environments or disciplines as it can be enriched with other languages. This flexibility has opened several times the question of opening it for non-SSH domains. While the consortium has noticed an interest from others, our experience has shown that it is better to have dedicated tools for a specific community. This is why all the design and the components of the GoTriple platform have been thought to be reshaped for new perspectives and reused. Taking this into account, instead of having one GS platform, we could have linked discovery platforms in different languages and disciplines which can be interoperable to improve users’ experience. There is a wish to see this platform as a first step in a more general process. 

## Data availability

### Underlying data

All data underlying the results are available as part of the article and no additional source data are required.

### Extended data

The interview data mentioned during the current study cannot be sufficiently de-identified and therefore cannot be made publicly available, due to ethical considerations. Please also note that the authors plan to use the interview data as core, underlying data in another future publication. For more information, please contact the corresponding authors.

## Ethics and consent

For the user research, ethical approval was granted by the Abertay University Ethics Committee (EMS1532). Written informed consent was obtained from all the participants in the user research.
