# Supplementary material for: Defining discovery: Is Google Scholar a discovery platform? An essay on the need for a new approach to scholarly discovery
Source: Open Res Eur. 2022 Jun 7;2:28. Originally published 2022 Mar 2. [Version 2] doi: 10.12688/openreseurope.14318.2 (PMC10445934; doi:10.12688/openreseurope.14318.2)
Supplement: Peer review history

# Peer review: Reviewer response for version 2

**DOI:** 10.21956/openreseurope.16066.r29483

**Published:** 2022-07-21

**Authors:** Jefferson Pooley

**Affiliations:** 1 Muhlenberg College, Allentown, PA, USA

Thanks to the authors for this thorough revision, which has addressed to this reviewer's satisfaction the facets of the earlier version that could be clarified, extended, or improved. It is an excellent paper that introduces GoTriple as a community-owned, SSH-focused alternative to Google Scholar while engaging with the literature on discovery in general and search engines in particular--with a particularly sophisticated treatment of discovery.

Is the argument persuasive and supported by appropriate evidence?

Partly

Is the work clearly and cogently presented?

Partly

Is the topic of the essay discussed accurately in the context of the current literature?

Yes

Does the essay contribute to the cultural, historical, social understanding of the field?

Yes

Reviewer Expertise:

Scholarly communication, academic publishing

I confirm that I have read this submission and believe that I have an appropriate level of expertise to confirm that it is of an acceptable scientific standard.

# Peer review: Reviewer response for version 1

**DOI:** 10.21956/openreseurope.15447.r28680

**Published:** 2022-03-29

**Authors:** Jefferson Pooley

**Affiliations:** 1 Muhlenberg College, Allentown, PA, USA

The article provides a cogent introduction to the GoTriple platform, with Google Scholar's limitations as the orienting foil. Limitations of GS around its indexing limitations (particularly around datasets and preprints), black box algorithm, and non-compliance with FAIR are used to set up a discussion of GoTriple, including the background of the TRIPLE project and the GoTriple design process. The core claim is that GoTriple provides an alternative approach, one that adheres to open data and open science principles, linguistic diversity, and transparency. An additional, if somewhat implicit, claim is that the design process, by consulting extensively with SSH academics, represents the expressed needs of the community in a way that commercial search engines like GS do not match. The paper includes an interesting discussion of the concept of "discovery" in a way that arguably extends the literature on this important, rarely defined, research practice.

 The article is coherent and well-organized overall. The authors chose to structure their paper as a rejoinder of sorts to GS; as a result, the first half is devoted to documenting the limitations of GS. This is a defensible approach, mainly because of documented researcher reliance on GS for research discovery. GS is the 800-pound gorilla, so to speak, so framing the paper around its shortcomings works well to set up the GoTriple alternative. 

 There are areas for improvement. More substantive issues are listed first, with lesser issues addressed second.

- There is a tension between discussing discovery across academia, on the one hand, and within SSH specifically, on the other. GS covers, or claims to cover, all research fields, while GoTriple is tailored to the SSH fields. This tension is partly resolved by the point, raised in the plain language summary especially, that SSH is especially poorly served by GS. However, the bulk of the treatment of GS in the actual paper is not oriented to GS's SSH limitations in particular. The authors should elaborate on the plain language summary's points about SSH in particular in the "Why Google Scholar is not a complete discovery platform" section, to include issues like poor book-chapter indexing. More broadly, the SSH specificity of GoTriple should be more directly addressed; by design, it is not a "complete discovery platform" either. There is an opportunity, particularly in the conclusion, to discuss the need for non-SSH supplementation to GoTriple, perhaps on the GoTriple model of extensive discussion with working academics, etc.
- A second area where the paper might better acknowledge GoTriple's particularity is in its European focus and origins. The paper notes this, but the European facet is in some tension with the "complete discovery platform" rhetoric. This is especially important because of the North-South inequality in scholarly communication more broadly. The relatively privileged geography, and still parochial scope in regional terms, should be acknowledged and openly grappled with. Is GoTriple's aim to be global in scope, within SSH? Is there openness to including non-European languages in its design? If not, this limitation should be acknowledged and addressed, perhaps with the claim that GoTriple's approach could be replicated elsewhere. The issue is that the paper's critique of GS rests, in part, on its English-language dominance; the less prominent critiques of Web of Science and Scopus make a similar point, if more so. One major line of criticism of the latter, in particular, is that they favor the Global North, especially the North Atlantic inclusive of North American and Northern Europe, especially outside the German-language area. While GoTriple is certainly challenging some of that, in particular the hegemony of English, it is still a European project of the Global North. Any "complete discovery platform" will need to more proactively include research and researchers beyond the rich West. Perhaps GoTriple is designed with this intent in mind, but in the current draft, there is little discussion of these global asymmetries.
- The paper places relatively little emphasis on the contrast between proprietary, for-profit services like GS, Scopus, etc., on the one hand, and GoTriple, on the other. Much more attention might be paid to this crucial difference, which is not only about adherence to the latest open data standards or ongoing innovation, but instead to core principles of community-owned and -led scholarly infrastructure. Here is an opportunity to underline what is one of the most attractive features of GoTriple: that scholars and the public own, fund, and direct it, vs. the commercial system that supplies most alternatives. Too much of the discussion, arguably, is focused on usability and quality of results, with neglect of the core community-control of infrastructure point. In this respect, the discussion on page 9 of open infrastructure is a bit oddly placed. The great bulk of the discussion is devoted to funding challenges, which (1) begs the question of how GoTriple will be funded going forward and, more importantly, (2) does not make (much) of the crucial *positive* case for open infrastructure like GoTriple.
- The actual description of what GoTriple does appears very late in the paper. It makes sense to center the early sections on GS's limitations. However, the paper arguably takes too long to provide an overview of GoTriple, partly because the more abstract discussion of "What is discovery?" as well as the subsequent discussion, come first. So it is not until page 9 that the reader learns more about the GoTriple alternative. Perhaps a short section after the GS criticism section could provide an overview earlier. 

Here are some smaller issues that could be addressed:

- Is it worth raising the commercial publishers' ambition to use GetFTR and ScienceDirect (Elsevier) as a competitor to Google Scholar and ResearchGate?
- Is it worth mentioning that Google could at any time move to monetize GS, along the surveillance capitalism lines of its main search engine?
- There is, occasionally, a tension between (1) emphasizing the black box nature of GS's algorithms and (2) stating with confidence what GS prioritizes (e.g., citations)
- There is a discrepancy between the plain language summary and the paper itself. The summary makes a stronger case that GS excludes materials ("Some platforms tend to choose materials that are useful only to the majority"). The discussion contradicts this, saying that—yes, GS excludes datasets, book reviews—but noting evidence that it is quite comprehensive within its stated aims about published research. The issue raised in the critique-of-GS section along these lines—in addition to the data+preprint, etc, exclusions—is that the algorithm upranks cited and English-language results. But this is different than 'choosing material that is useful only to the majority'. I suspect the intended meaning here is that long-tail works are hard to discover — but exclusion isn't the right word.
- Mentioning the relatively bad indexing of book chapters seems to be an opportunity, re SSH.
- The paper references March 2022 for the rollout; given the date, this could be updated.

Is the argument persuasive and supported by appropriate evidence?

Partly

Is the work clearly and cogently presented?

Partly

Is the topic of the essay discussed accurately in the context of the current literature?

Yes

Does the essay contribute to the cultural, historical, social understanding of the field?

Yes

Reviewer Expertise:

Scholarly communication, academic publishing

I confirm that I have read this submission and believe that I have an appropriate level of expertise to confirm that it is of an acceptable scientific standard, however I have significant reservations, as outlined above.

# Response

**Published:** 2022-05-30

**Authors:** Francesca Di Donato

Thank you for your review. We reply here to the main issues you raised first, and then to the less important ones:

1. Additional text has been added to the section “Why GoogleScholar is not a complete discovery platform” (at the end) elaborating better on: 1) the connections between google scholar and SSH research and on 2) the specificities of GoTriple. On point 1) there is not much literature on the subject, but we have reviewed 3 relevant items now. Existing literature on GS and SSH specifically focus again on the comparison between GS and established citation databases. This literature shows that GS is more inclusive than the other services, at the price of less “Quality” in the material. In your comment there was a pointer to the fact that GS does not index book-chapters well (which of course are important publication items in SSH), however the literature does appear to say that GS is better at indexing e.g. chapters or dissertations if compared to databases. Nonetheless we added a paragraph pointing still to the limits of GS and arguing for the need of a specific and even more inclusive solution for SSH. This is now connected directly with the focus of GoTriple in codesigning together with the SSH community the solution, as well as on the much more encompassing idea of discovery focusing not only on publications but also on the discovery of people, projects etc.
2. About the “complete discovery platform”: GoTriple aims for a global coverage of SSH scientific fields. “Complete” here is intended as in the complete discovery experience GoTriple offers in terms of openness, findability and coverage in comparison with GS which limits users in their discovery process (silo effect, lack of reusability).
  Furthermore, the TRIPLE project is European-funded and thus naturally has a stronger focus on European data sources at the moment. But the inclusion of Spanish, Portuguese, Greek and Croatian among the 9 languages that were chosen reflects the objective to reach out to and include not only stakeholders from the rich west but also South-European and non-European research actors. We acknowledge that the sources included in the platform at the moment are more focused on Europe and the Global North, which mostly depends on our data providers and the data sources that we are using. However, it is intended to extend the available data sources beyond the project duration to include sources from, e.g., the Middle- and South Americas (Spanish, Portuguese).
  While European languages have been prioritised, the GoTriple platform is designed to enable the inclusion of non-European languages. For new languages to be added, a step-by-step process combined with an evaluation of the usage/demand for the additional language will be performed.
  GoTriple is presented as a complete discovery platform in terms of the experiences it provides: not only textual results, but also recommendations, visual results (graphs) or even crowdfunding to improve the discovery of SSH domain for citizens. It is complete also regarding the whole SSH disciplines it targets. However, by nature, it is not for all the scientific disciplines. In this regard and based on your comment, we changed “complete” by “open and inclusive” to avoid any confusion on this topic.
3. In contrast to proprietary, for-profit services (like Google Scholar, Scopus, etc.), GoTriple goes further than adhering to the latest open data standards. In fact, the Gotriple platform embraces the core principles of a community-owned and community-led infrastructure. An attractive feature of GoTriple is that its users own it, fund it, and direct it (ie. scholars and the public).
  The core community control of the open infrastructure is mirrored in the envisaged governance model for the platform (figure below). OPERAS will provide the sustainability of the service and will include a GoTriple Committee (composed of members of OPERAS - new  memberships from TRIPLE partners are expected to come about in the last year of the project). The Committee will be divided in two subgroups: the User Engagement subgroup and the Data and Tools subgroup. This model will ensure that user feedback and needs are taken into account and that the community is in control when it comes to the further development and direction of the platform, e.g. which 3rd party tools are integrated. This work is currently done and planned for the last year of the project. 
  The aim is to create a reusable, community-owned infrastructure that is a public good. All software is developed following open standards and is available under permissive open source licenses. The advantage of the open source approach is that the software can easily be migrated to other systems and thus lock-in effects are avoided.
  In addition, GoTriple is developed following open standards. GoTriple’s data and source code are available under open licenses where possible. The advantage of this approach is that parts of the platform can easily be reused and that the system can easily be migrated. Proprietary lock-in effects are thus avoided. Making its enriched, high-quality data available means that not only GoTriple users will benefit from a better discovery experience, but that GoTriple will also play an important role in improving the data quality for SSH within the whole digital open science ecosystem.
4. Indeed, the following paragraph has been added at the end of section 1 *: “In light of GS's limitations the TRIPLE project built a discovery platform, GoTriple, which aims to provide a service to discover SSH resources (data & publications, profiles of researchers and scientific projects) with a multilingual perspective. By linking these resources together in one platform, the user's experience and the ability for researchers and society at large to discover resources for their specific purposes should be much improved. The TRIPLE project has worked with stakeholders, from across SSH disciplines and wider, from the very start to ensure that the platform will take into account their needs for a discovery platform. In doing so, all aspects discussed throughout this paper that constitute effective discovery platforms have been taken into consideration. Moreover the GoTriple platform enables users to discover and reuse open scholarly SSH resources in nine European languages which are currently scattered across local and discipline-specific repositories, it also provides opportunities for researchers and projects to connect across disciplines and languages. A set of innovative tools and services are integrated in the platform to support research and enable, among others, the visualisation of search results, web annotation, personalised recommendations and social networking practices. In line with the aim to connect researchers and projects in the SSH, the platform allows users to explore new ways of funding research such as crowdfunding. The presentation of the GoTriple discovery platform and its features will be deepened further in the article (see section 4).”*
  Our answers to smaller issues are the following:
5. This has been discussed a lot but the authors thought that there is a risk to develop another topic and not staying focused on the heart of the article.
6. This point has been précised in section 3 of the paper.
        
7. We have tried to partially solve this tension. However, even the Google’s own words are confused on this topic as we can show here: https://scholar.google.com/intl/de/scholar/about.html): "Google Scholar aims to rank documents the way researchers do, weighing the full text of each document, where it was published, who it was written by, as well as how often and how recently it has been cited in other scholarly literature."
8. This point has been addressed based on the suggestions.
9. This point has been precised in the paper.
10.  Addressed.

# Peer review: Reviewer response for version 1

**DOI:** 10.21956/openreseurope.15447.r28677

**Published:** 2022-03-28

**Authors:** Roberta Bracciale, Junio Aglioti Colombini

**Affiliations:** 1 Department of Political Science, University of Pisa, Pisa, Italy

This article deals with a complex and cogent theme for researchers being the consultation of literature and academic sources. In a time of information overload, finding research material can be extremely challenging if web search engines for scholarly literature are inadequate.

 Authors describe in particular the deficiencies and the limits of Google Scholar (GS), the biggest free platform used by researchers. Managing a solid theoretical framework, authors underline the problematic aspects of GS, addressing the limits of the affordances (e.g. the absence of research filters) as well as the lack of transparency of the service algorithms and how this makes GS “unlikely to be the exemplar of equality and transparency” (p.4). To address this topic, the authors use accurately the literature that had previously analysed these platforms demonstrating a systematic job review of the state of the art.

 In the initial discussion about citation databases, the authors briefly mention the impact that platforms can have on minorities (p.4, paragraph 4). We believe that further elaboration on this matter could benefit the article and make even more explicit how a well-structured bibliographic database could benefit the whole research environment.

 The assertions sustained by the authors about the urgency to rethink the current model are grounded in today’s context and based on actual problems that the qualitative interviews allowed to emerge. As a result, the development of the platform GoTriple takes into consideration the specific needs of the current scientific knowledge and offers solutions that are tailored for the new generations of researchers, making the arguments persuasive, and supported by evidence and concrete needs. The work is overall well written, and the contents are clearly organized.

 The paper contributes to deepening the matter of bibliographic platforms offering a way to approach this complex theme and some future implementations but also acknowledging the limits of a giant work that should be done cooperative as well as the impossibility to find a unique solution for everybody.

 We recommend indexing in its current form.

Is the argument persuasive and supported by appropriate evidence?

Yes

Is the work clearly and cogently presented?

Yes

Is the topic of the essay discussed accurately in the context of the current literature?

Yes

Does the essay contribute to the cultural, historical, social understanding of the field?

Yes

Reviewer Expertise:

Digital Sogiology

We confirm that we have read this submission and believe that we have an appropriate level of expertise to confirm that it is of an acceptable scientific standard.
